# Functionalized Glutathione on Chitosan-Genipin Cross-Linked Beads Used for the Removal of Trace Metals from Water

**DOI:** 10.1155/2020/4158086

**Published:** 2020-09-14

**Authors:** Samira R. Akaji, David Dewez

**Affiliations:** Laboratory of Environmental & Analytical Biochemistry of Contaminants, Department of Chemistry, University of Quebec in Montreal, Montréal, C.P. 8888 Succursale Centre-Ville, Canada

## Abstract

Functionalized glutathione on chitosan-genipin cross-linked beads (CS-GG) was synthesized and tested as an adsorbent for the removal of Fe(II) and Cu(II) from aqueous solution. The beads were characterized by several techniques, including Fourier-transform infrared spectroscopy (FTIR), thermogravimetric analysis (TGA), CNS elementary analysis, scanning electron microscopy (SEM), and atomic force microscopy (AFM). The effect of several parameters such as the pH, the temperature, and the contact time was tested to optimize the condition for the adsorption reaction. The beads were incubated in aqueous solutions contaminated with different concentrations of Fe(II) and Cu(II) (under the range concentration from 10 to 400 mg·L^−1^), and the adsorption capacity was evaluated by inductively coupled plasma optical emission spectrometry (ICP-OES). The adsorption equilibrium was reached after 120 min of incubation under optimal pH 5 for Fe(II) and after 180 min under optimal pH 6 for Cu(II). According to the *Langmuir* isotherm, the maximum adsorption capacities (*q*_max_) for Fe(II) and Cu(II) were 208 mg·g^−1^ and 217 mg·g^−1^, respectively. Our results showed that the adsorption efficiency of both metals on CS-GG beads was correlated with the degree of temperature. In addition, the adsorption reaction was spontaneous and endothermic, indicated by the positive values of Δ*G*^0^ and Δ*H*^0^. Therefore, the present study demonstrated that the new synthesized CS-GG beads had a strong adsorption capacity for Fe(II) and Cu(II) and were efficient to remove these trace metals from aqueous solution.

## 1. Introduction

The development of urbanization including industrial and agricultural activities has been the main source of metal pollution in the environment. The continuous release of trace metal elements in water reservoir may represent a risk of toxicity for natural ecosystems and human health, since these metals are nonbiodegradable and can be bioaccumulated in aquatic organisms [1–3]. Therefore, water pollution by metals is still a global problem for environmental quality and public health. In particular, Fe(II) and Cu(II) are both nutrients at very low concentrations involved in many biological functions such as cofactors for enzymatic activities but become toxic at high concentrations [4–6]. According to the WHO standards, the acceptable limits of Fe(II) and Cu(II) in drinking water are 0.3 mg·L^−1^ and 2.0 mg·L^−1^, respectively [7]. Indeed, water with high Fe(II) content can represent a substrate for the development of bacterial contamination in the ducts [8]. It was reported that chronic exposure to Fe(II) may lead to adverse health effects, such as impaired hematopoiesis [9]. In addition, Cu(II) overload can induce adverse health effects, such as keratinization, gastrointestinal, liver, or kidney disorders [10–12].

To preserve water quality, different remediation strategies have been developed to control the concentration of trace metal elements in a freshwater reservoir. Until now, remediation methodologies have included chemical precipitation, reverse osmosis, coagulation and flocculation, oxidation, electrochemical treatment, ion exchange, solid-phase extraction, adsorption on activated carbon, and biosorbents [1, 2, 13]. In particular, the use of adsorbents showed to be the most economical and effective method for the removal of metals from aqueous solution, without producing toxic sludge (by-products of treatment). Several types of adsorbent were studied such as clay mineral, activated carbon, porous carbon, porous carbon loaded with ZnO nanoparticles, nanomaterials, chitosan and modified chitosan, carbon nanotubes coated with alumina, zeolites, natural polymers, and mesoporous treated fish waste [14–29]. Among natural polymer adsorbents, the chitosan (CS) represented a “green” and eco-friendly compound for the adsorption of metals in solution, because of its hydrophilicity, biocompatibility, biodegradability, and biofunctionality properties [30]. The synthesis of CS is usually obtained by the alkaline deacetylation of chitin, a natural polysaccharide found in the exoskeletons of crustaceans like shrimps, crabs, prawns, and lobsters [31, 32]. To increase the stability and the capacity of CS beads, cross-linking agents are used such as glutaraldehyde, epichlorohydrin, and ethylene glycol diglycidyl ether, leading to the formation of a three-dimensional network [33–36]. However, these chemicals are known to be toxic to living organisms. In fact, the cytotoxic, mutagenic, and carcinogenic properties of glutaraldehyde were previously demonstrated [37, 38].

It is well known that the adsorbent property of CS is due to the presence of amine (–NH_2_) (acetamide or primary amino) and hydroxyl (–OH) groups, providing coordination sites for the complexation of metals. To improve the maximal adsorption capacity of CS, the design of chelating CS-based resin has been the subject of many studies [21, 30, 39]. Previously, different sulfur compounds were added to CS, such as dithiocarbamate [40], thiourea or dithiooxamide [41], and mercapto acetic acid [42]. Recently, the ligand cysteine was used for the functionalization of CS to bind more efficiently metals in aqueous solutions [43]. However, other thiol compounds were not investigated and should be tested to determine the efficiency of new complexes. In particular, phytochelatins (PCs) are well-known thiol polypeptides, consisting of a chain of 2 to 11 units of glutathione (*γ*-L-glutamyl-L-cysteinylglycine, GSH). In plant cells, these compounds are chelators involved in the transport of metals into the vacuole, maintaining the homeostasis of intracellular concentration of metals [44]. Since the synthesis of PCs is costly, its repetitive subunit GSH can represent a more practical alternative. In addition, the GSH is composed of three amino acids as *γ*-L-glutamyl-L-cysteinylglycine, providing the functional groups –COOH, –SH, and–NH_2_ for the complexation of metals [45].

The main objective of this study was to design a new complex CS-genipin by covalent cross-linking reaction and functionalized with GSH to obtain a strong adsorption property for metals in solution. It is known that genipin is a natural cross-linking agent derived from geniposide, a compound extracted from the fruit *Gardenia jasminoides* Ellis (Rubiaceae). This plant was traditionally used by Asian populations as a medicinal herb and food coloring, and its toxicity was demonstrated to be a thousand times (5000–10000) lower than glutaraldehyde. It was reported that genipin can make covalent bonds with amino groups of proteins and biopolymers such as CS [46–48]. For these reasons, we used in this study the genipin as a cross-linking agent to make the beads. The new beads were characterized by Fourier-transform infrared spectroscopy (FTIR), thermogravimetric analysis (TGA), CNS elementary analysis, scanning electron microscopy (SEM), and atomic force microscopy (AFM). In addition, we investigated their adsorption capacity in aqueous solution for both Fe(II) and Cu(II) ions, and the quantification was performed by inductively coupled plasma optical emission spectrometry (ICP-OES). The effect of different parameters (variables) was also investigated on the adsorption rate, such as the pH, the temperature, the reaction time, and the adsorption isotherms. Therefore, this study determined the optimal condition for the adsorption of Fe(II) and Cu(II) ions on these new CS-genipin cross-linked beads with functionalized GSH (CS-GG).

## 2. Material and Methods

### 2.1. Material and Reagents

The CS (Kitomer™, MW 1600 kDa, 85–89% D Ac) was obtained from Marinard Biotech (QC, Canada). The genipin was purchased from Challenge Bioproducts Co., Ltd. (Taiwan). The reduced form of glutathione at high purity was provided by Bio Basic Inc. (ON, Canada). The chemicals CuCl_2_, FeCl_2_.4H_2_O, and anhydrous ethanol were purchased from Sigma–Aldrich Chemical Co. All chemicals were of analytical grade and used as received without any additional purification. All subsequent experiences were carried out with Nanopure water provided by a Barnstead Nanopure water purification system (ThermoFisher Scientific).

### 2.2. Preparation of Chitosan-Based Beads

#### 2.2.1. Preparation of Bead-Forming Solution

A mixture of CS (5 g) and aqueous acetic acid (250 ml, 0.1 % (v/v)) was stirred at 150 rpm for 24 h at room temperature to ensure total solubility of the CS. Then, a simple ultrasound device (Fisher Scientific Model 505 Sonic Dismembrator) was used to remove air bubbles in the solution before forming the beads.

#### 2.2.2. Gelification of Chitosan Beads under Alkaline Condition

The previous gelatinous mixture was released through a syringe needle (1 mm in diameter) into 1 M NaOH solution with 70% alcohol, in which the CS was precipitated immediately to form gelatinous spherical beads. The obtained CS beads were thoroughly washed with Nanopure water followed by ethanol (70%) until neutrality. The beads were kept in 0.01 M of sodium phosphate buffer (pH 7.0) at 4°C until further use. Before using them, the beads were generously washed three times with ethanol (70%) and three times with Nanopure water.

#### 2.2.3. Formation of Chitosan-Genipin Cross-Linked Beads

The CS beads obtained in the previous step did not have satisfactory mechanical properties. To improve them, the beads were cross-linked with genipin to reinforce the structure of the covalent bonds between CS and genipin. An amount of 50 g of CS beads was put into a cross-linking solution containing 5.6 mg of genipin (0.05% of CS weight). The suspension was moderately stirred at room temperature during 48 h. Then, the cross-linked beads were rinsed with ethanol (70%) and Nanopure water to remove the excess of cross-linking solution.

#### 2.2.4. Glutathione Functionalization on Chitosan-Genipin Cross-Linked Beads

An amount of 50 g of CS-genipin cross-linked beads was added to 200 mL of a solution containing 2 mg·mL^−1^ of GSH and 0.1 mM of genipin, which were then incubated at 25°C during 24 h under a mildly stirring. Before decantation, beads were rinsed with Nanopure water to remove the excess of by-products and other impurities. With this method, we obtained beads functionalized at their surface with a thin layer of GSH (CS-GG).

### 2.3. Characterization of the Beads

#### 2.3.1. Fourier-Transform Infrared Spectroscopy Analysis

Fourier-transform infrared (FTIR) spectra were recorded using a Spectrum One spectrophotometer (Thermo Scientific Nicolet 4700 iTR) equipped with a universal attenuated total reflectance (UATR) device. The analysis was performed in the spectral region (4000–500 cm^−1^) with 32 scans recorded at a 16 cm^−1^ resolution.

#### 2.3.2. Thermogravimetric Analysis

Thermogravimetric analysis (TGA) was done on native CS and CS-GG beads by using a TGA/MS analyser (TGAQ5000 Discovery MS). Experiments were performed under a dynamic argon atmosphere flowing at a rate of 15 mL·min^−1^ and at a heating rate of 2.5°C·min^−1^.

#### 2.3.3. Microscopy Measurements

Scanning electron microscopy (SEM) was performed on CS and CS-GG beads to characterize their topography by using a field emission scanning electron microscope JEOL (model: JSM-7600F). In addition, atomic force microscopy (AFM) was used to analyze the surface topography of CS-GG beads with a Bruker MultiMode 8 AFM system.

#### 2.3.4. CNS Elementary Analysis

The elementary analysis was carried out by using a Carlo Erba Instruments NA2500 series elemental analyser. The samples were dried, and 20 mg was put in tin capsules to measure the percentage of C, N, and S elements.

#### 2.3.5. Adsorption Experiments

All metal adsorption studies were performed in batch experiments by using 125 mL Erlenmeyer flasks containing 50 mL of trace metal solution and 1 g of CS-GG beads. Stock solutions were prepared at 500 mg·L^−1^ of Fe(II) and Cu(II). The experiments were performed under the range concentration from 10 to 400 mg·L^−1^ and different pH conditions (from 1 to 7), which was adjusted by using HCl/NaOH solutions. The kinetics were studied at the optimum pH of 5.0 and 6.0 for Fe(II) and Cu(II), respectively, and under the constant temperature of 20°C. The analysis was carried out in triplicate at regular time intervals (10 to 400 min). Three Erlenmeyer flasks were prepared (as mentioned before) for each time interval, from which aliquots (2 mL) were taken to determine the quantity of metal ions adsorbed on CS-GG beads.

Equilibrium isotherm studies were carried out with different concentrations of metal ions (10 to 400 ppm) at different temperatures. The thermodynamic parameters were determined by changing the temperature to 20°C, 30°C, and 40°C in a temperature-controlled shaking incubator (Infors HT Multitron Pro thermostatic). The flasks were agitated on a shaker at 150 rpm during 2 h. After the incubation, the solution was filtered and the concentration of metal ions was determined using inductively coupled plasma optical emission spectrometry (ICP-OES, Agilent model 5100, Agilent Technologies, USA). All experiments were performed in triplicate, and the average values were estimated. The quantity of metal ions was estimated per unit mass of the adsorbent according to the following equation:

(1) $$\begin{array}{c} q_{e}=\frac{\left(C_{0}-C_{e}\right)}{m}v, \end{array}$$

where *q*_*e*_ is the adsorbed amount of metal ions per unit mass of the adsorbent (mg · g^−1^) at equilibrium, *m* (g) is the weight of the CS-GG beads, *C*_0_ is the concentration of Fe(II) and Cu(II) before the adsorption, *C*_*e*_ (mg · L^−1^) is the concentration of Fe(II) and Cu(II) after equilibrium with CS-GG beads, and *v* (L) is the volume of the solution.

#### 2.3.6. Adsorption Isotherms

Equilibrium adsorption isotherms were used to determine the adsorption mechanism and capacity for metals. Some well-known ones are *Langmuir*, *Freundlich*, *Temkin*, *Redlich–Paterson*, *Dubinin–Radushkevich*, and *Sips* equations [49–51]. The adsorption isotherms of *Langmuir* and *Freundlich* models were used to describe the adsorption equilibrium of Fe(II) and Cu(II) ions on CS-GG beads. The experiments were performed at different temperatures (20, 30, and 40°C).

The *Langmuir* isotherm assumed that the mechanism of metal ions' adsorption process took place as a monolayer on the surface of the adsorbent (beads). The linear form of *Langmuir* isotherm was expressed by the following equation:

(2) $$\begin{array}{c} \frac{C_{e}}{q_{e}}=\frac{1}{q_{\max}}C_{e}+\frac{1}{K_{L}\cdot q_{\max}}, \end{array}$$

where *C*_*e*_ is the equilibrium concentration of remaining metal ions in the solution (mg·L^−1^), *q*_*e*_ is the amount of metal ions adsorbed per mass unit of adsorbent at the equilibrium (mg·g^−1^), *q*_max_ (constant) is the amount of metal ions for a complete monolayer (mg·g^−1^), and *K*_*L*_ is the *Langmuir* constant related to the affinity of binding sites (L·mg^−1^), representing a measure of the adsorption energy. *q*_max_ and *K*_*L*_ were evaluated from the intercept and the slope of the linear plot of the experimental data of *C*_*e*_/*q*_*e*_ versus *C*_*e*_, respectively. To determine the adsorption affinity of Fe(II) and Cu(II) ions on CS-GG beads, the separation factor *R*_*L*_ was determined. This factor was calculated by the following equation:

(3) $$\begin{array}{c} R_{L}=\frac{1}{\left(1+K_{L}\cdot C_{0}\right)}, \end{array}$$

where *R*_*L*_ is a dimensionless separation factor, indicating the shape of the isotherm, *C*_0_ is the initial concentration of metal ions, and *K*_*L*_ is the *Langmuir* constant. The tendency of the adsorption process was indicated by *R*_*L*_ values. When *R*_*L*_ > 1, the isotherm was unfavorable; for *R*_*L*_ = 1, the isotherm was linear; when *R*_*L*_ < 1, the isotherm was favorable; for *R*_*L*_ = 0, the reaction was irreversible.

The *Freundlich* isotherm assumed that the mechanism of metal ions' adsorption process did happen as a multilayer on a heterogeneous surface of the adsorbent (beads), given by the linear form of the following equation:

(4) $$\begin{array}{c} \ln\;q_{e}=\frac{1}{n}\left(\ln\;C_{e}\right)+\ln\;K_{F}, \end{array}$$

where *K*_*F*_ and *n* (or 1*/n*) are both a constant, indicating the adsorption capacity (mg·g^−1^) and the intensity (degree of surface heterogeneity), respectively. The fitting plots based on *Langmuir* and *Freundlich* models for the adsorption of Fe(II) and Cu(II) are presented in Figures S2(a) and S2(b) and Figures S3(a) and S3(b).

#### 2.3.7. Adsorption Kinetics

The adsorption on a solid surface can be controlled by several steps such as the boundary layer (film) or external diffusion, the pore diffusion, the surface diffusion, and the adsorption on a pore surface. We investigated the adsorption process of Fe(II) and Cu(II) ions on CS-GG beads by correlating our results with different kinetic models: the pseudo-first-order [52], the pseudo-second-order [53], and the intraparticle diffusion [54].

The linear form of the pseudo-first-order equation was as follows:

(5) $$\begin{array}{c} \log\left(q_{e}-q_{t}\right)=\log\;\;q_{e}-\frac{k_{1}}{2.303}t, \end{array}$$

where *k*_1_ is the pseudo-first-order rate constant (min^−1^) of adsorption and *q*_*e*_ and *q*_*t*_ (mg·g^−1^) represent the amount of metal ions adsorbed at the equilibrium and at the time *t* (min), respectively. The straight-line plots of log (*q*_*e*_−*q*_*t*_) versus *t* were used to determine *k*_1_ and the correlation coefficient *R*^2^, which is shown in Figures S4(a) and S4(b).

Furthermore, the linear form of the pseudo-second-order equation was as follows:

(6) $$\begin{array}{c} \frac{t}{q_{t}}=\frac{1}{k_{2}\cdot q_{e}^{2}}+\frac{1}{q_{e}}t, \end{array}$$

where *k*_*2*_ is the pseudo-second-order rate constant of adsorption (g mg^−1^·min^−1^). The value of 1*/q*_*t*_ was calculated from the experimental results and plotted versus 1*/t* (min^−1^) to obtain the biosorption rate constant (*k*_2_), shown in Figures S5(a) and S5(b).

Kinetics data were also fitted with the intraparticle diffusion model, which considers that if the rate-limiting step was the intraparticle diffusion, then the amount adsorbed *q*_*t*_ (mg·g^−1^) at any time *t* (min) should be directly proportional to the square root of the contact time *t*. This model was defined by Weber and Morris [54], and the equation was given as follows:

(7) $$\begin{array}{c} q_{t}=k_{i\;d}\cdot t^{0.5}+C, \end{array}$$

where *q*_*t*_ (mg·g^−1^) is the amount adsorbed at the equilibrium time *t* (min), *k*_*id*_ (mg · g^−1^·min^−1^) is the intraparticle diffusion rate constant, and *C* is the intercept of the plot of *q*_*t*_ against *t*^0.5^, providing information about the thickness of the boundary layer. The greater the value of *C* is, the greater the effect of the boundary layer on the adsorption is [51].

#### 2.3.8. Thermodynamic Analysis

Thermodynamic parameters including the standard Gibbs energy change (Δ*G*°), the enthalpy change (Δ*H*°), and the entropy change (Δ*S*°) of the adsorption reactions of Fe(II) and Cu(II) were determined by using the following equations:

(8) $$\begin{array}{c} \Delta G^{{}^{\circ}}=-RT\;\ln\;K_{c},\\ K_{c}=\frac{1000q_{e}}{C_{e}}, \end{array}$$

where *R* (8.314 × 10^−3^ kJ·mol^−1^ · K^−1^) represented the molar gas constant, *T* is the temperature (*K*), and *K*_*c*_ is the distribution coefficient at different temperatures (20, 30°C, and 40°C). The equilibrium constant *K*_*c*_ was related to the change of the Gibbs free energy process. To make *K*_*c*_ dimensionless, *q*_*e*_ was multiplied by 1000 before taking the logarithm [55–57]. In addition, the change in the Gibbs free energy was related to the change sof enthalpy and entropy at a constant temperature, according to the following equation:

(9) $$\begin{array}{c} \Delta G^{{}^{\circ}}=\Delta H-T\Delta S^{{}^{\circ}}. \end{array}$$

Values of Δ*H*° and Δ*S*° were calculated from the slope and intercept of the linear plots of −Δ*G*° versus *T* (Figure S1). The slope and intercept of the plot gave the Δ*S*° and the −Δ*H*° values, respectively.

## 3. Results and Discussion

### 3.1. Characterization of Polymer Samples

**Figure 1:**
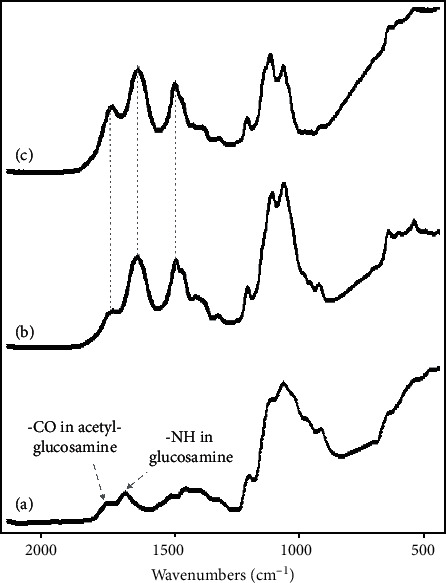
FTIR spectra of CS (a), CS-genipin (b), and CS-GG beads (c). For more details, see Materials and Methods.

The obtained spectra of CS, CS-genipin, and CS-GG beads determined by FTIR analysis are presented in Figure 1. The CS selected in the present study possessed a DDA of 89%, indicating 89% of glucosamine and 11% of acetyl-glucosamine. For this reason, the native CS absorption spectrum showed two specific absorption bands (Figure 1(a)), the first one located at 1650 cm^−1^ was related to acetyl-glucosamine, and the second one at 1550 cm^−1^ was attributed to the primary amine (planning vibration) from glucosamine. This confirmed the presence of residual N-acetyl groups in CS, as it was previously reported [58]. When CS was cross-linked with genipin, the absorption bands at 1655 and 1575 cm^−1^ disappeared (Figure 1(b)), and a new absorption band located at 1640 cm^−1^ was related to the carbonyl from genipin. In addition, the comparison of CS-GG and CS-genipin spectrum presented no significant differences (Figure 1(c)). However, the intensity of the absorption band at 1550 cm^−1^ appeared to be related to the carbonyl from genipin, and the absorption band from the carboxyl of GSH was significantly increased.

**Figure 2:**
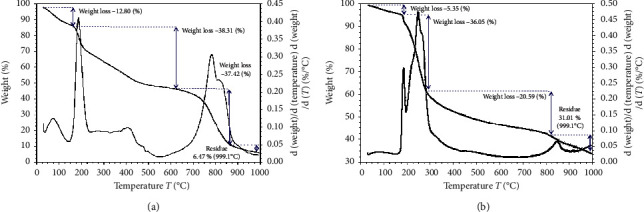
Thermogravimetric analysis of CS beads (a) and CS-GG beads (b).

The TGA analysis showed that thermogravimetric curves (TG) displayed three important phases for CS beads (Figure 2(a)) and CS-GG beads (Figure 2(b)). The weight loss of CS beads was 12.8% under the temperature range of 30°C–170°C, and this was attributed to the moisture in the beads. The TG curve showed a mass loss of 38.31% under 175°C–650°C, 37.42% under 675°C to 870°C, and 5.05% under 875°C to 999.1°C. However, the CS-GG beads were thermally more stable than CS beads, and the loss due to the moisture was 5.35% in the temperature range of 30°C–178°C. In addition, the TG curve showed a 36.05 and 20.59% weight loss under the range of 178°C–276°C and 290°C–825°C, respectively. These results were attributed to the splitting of the saccharide rings. Furthermore, the TG curve indicated a final weight loss for the CS beads of about 93.53%, while for CS-GG beads, it was about 68.99% at 999.1°C. These results confirmed the modification of the native CS beads by genipin cross-linking and the functionalization of GSH, giving the property to be more thermally stable. This can be explained by the formation of a rigid polymer network, resulting in a higher thermally stable composite.

The elemental analysis of carbon, azote, and sulfur was performed on the beads, and the weight ratio of C, N, and S for CS beads was found to be 40.48, 6.76, and 0%, respectively. In a previous study, similar values in CS were found for C, N, and S contents with 40.2, 7.41, and 0%, respectively [59]. Our results did suggest a high purity of the CS used. The proportions of C, N, and S contents in CS-GG beads were 68.57, 18.31, and 0.72%, respectively. In fact, the percentage of S was directly proportional to the amount of GSH functionalized into the beads.

**Figure 3:**
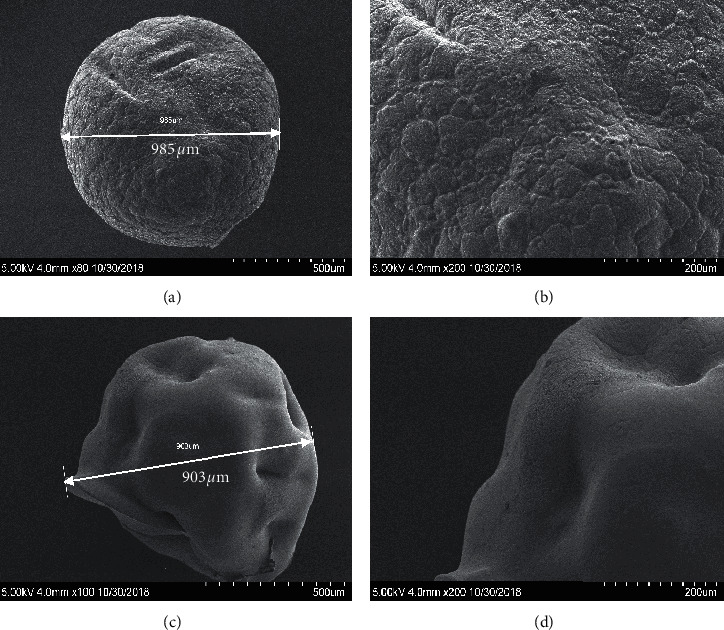
SEM photography showing the surface morphology of CS beads (a, b) and CS-GG beads (c, d).

**Figure 4:**
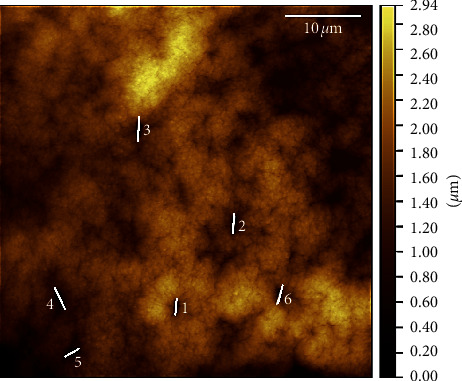
AFM image of CS-GG beads.

The obtained SEM photography showed the surface morphology of CS beads (Figures 3(a) and 3(b) and CS-GG beads (Figures 3(c) and 3(d). The micrographs presented the homogeneity of the adsorbent, and the presence or not of voids and aggregates onto the surface. In fact, the surface of CS-GG beads was more uniform than the surface of CS beads, indicating a better homogenization film of cross-linked chitosan. In addition, the AFM was used on CS-GG beads to obtain a detailed observation of the nanocomposite film surface at high resolution (Figure 4). The obtained AFM image of CS-GG beads revealed the presence of voids onto the surface.

### 3.2. Optimization of the Adsorption Capacity for CS-GG Beads

The effects of pH, temperature, contact time, initial concentration of metal ions in solution, and thermodynamic and adsorption kinetic parameters were investigated on the adsorption capacity of Fe(II) and Cu(II) on CS-GG beads.

#### 3.2.1. Effect of pH

**Figure 5:**
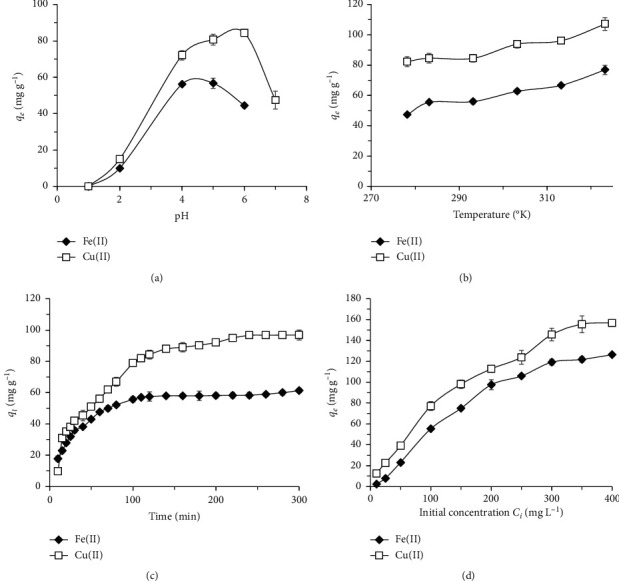
Effect of the initial pH (a), the temperature (b), the contact time (c), and the initial concentration of metal ions in solution (d) on the adsorption capacity of CS-GG beads for Fe(II) and Cu(II). The error bars represent the standard deviation of measurements for three sample runs (*n* = 3).

It is well known that the pH is one of the most important factors during the adsorption process, since it can affect the speciation and the solubility of metal ions, their concentration, the functional groups of the adsorbent, and the degree of ionization during the reaction [60–63]. In this study, adsorption experiments were conducted in the pH range of 2–6 for Fe(II) and 2–7 for Cu(II). However, Fe(II) and Cu(II) precipitated as insoluble hydroxides at pH > 6 and at pH > 7, respectively.  Figure 5(a) showed that increasing the pH values of the solution from 2 to 5 induced an increase of the adsorption capacity for Fe(II) and Cu(II). According to previous studies, when the pH of the medium was low, the high concentration of proton (H^+^) did occupy most of the adsorption-binding sites at the surface of the adsorbent (i.e., protonation of the amino group). The H^+^ did compete with metal ions, causing a decrease in adsorption efficiency [60–63]. In our study, the CS-GG beads were protonated and gained electrostatic properties (–COO^−^, –NH_3_^+^, –SH). However, it is most likely that the binding of divalent metal ions Fe(II) and Cu(II) was stronger than H^+^, since the electrostatic interaction between the pair of electrons on the nitrogen atom and the metal ion was stronger than H^+^. In addition, there was a mechanism of ion exchange or competitive adsorption between the metal ions and the H^+^ on the amino group [62, 63]. Moreover, the increase in the pH value induced a decrease in the protonation of the amine groups, facilitating the deprotonation of both carboxylic and –SH groups, and releasing more binding sites. This reaction might increase the coordination sites for metal ions on CS-GG beads and the formation of an inner-sphere complex by a surface chelation ion exchange [43, 62, 63]. This explanation agreed with our obtained results concerning the increase in the adsorption capacity of metal ions shown in Figure 5(a). Therefore, we found that the optimum pH for the adsorption of Fe(II) and Cu(II) onto CS-GG beads was 5 and 6, respectively.

#### 3.2.2. Effect of Temperature

The effect of temperature on the adsorption capacity of CS-GG beads for Fe(II) and Cu(II) was investigated under the range of 20°C to 40°C (Figure 5(b)). During these experiments, all the other parameters were kept constant at their optimum values, including a pH of 5 for Fe(II) and 6 for Cu(II), an adsorbent quantity of 0.03 g, and a contact time of 120 min. The results showed that the adsorption capacity increased from 47.26 to 76.87 mg·g^−1^, in the temperature range tested for Fe(II). Concerning Cu(II), the adsorption capacity increased from 82.26 to 107.11 mg·g^−1^ under the same range of temperature (Figure 5(b)). The enhancement in adsorption capacity was correlated with the increase in temperature. This can be attributed to a higher number of active surface sites available during the adsorption and to an increase in porosity and pore volume of the adsorbent [64]. Therefore, the correlation between the adsorption capacity and the temperature indicated that the adsorption reaction of Fe(II) and Cu(II) ions on CS-GG beads was endothermic.

#### 3.2.3. Thermodynamic Analysis

Thermodynamic parameters characterizing the adsorption reaction of Fe(II) and Cu(II) onto CS-GG beads were analyzed, such as the changes in Gibbs standard energy (Δ*G°*), in enthalpy (Δ*H°*), and in entropy (Δ*S°*), and the results were presented in Table 1. Based on thermodynamics, since the reaction did take place in an isolated system, the energy in the system could not be gained or lost, and the entropy change would be the only driving force [65]. Our results showed that the negative value of the Gibbs free energy (Δ*G*° < 0) increased in relation to the increase in temperature, indicating the probability and spontaneity of both Fe(II) and Cu(II) adsorption reactions onto CS-GG beads. The positive value of the enthalpy change (Δ*H*° > 0) of Fe(II) and Cu(II) adsorption reactions indicated the endothermic property of these reactions. In addition, the positive value of the entropy change (Δ*S° > 0*) indicated that the number of species at the solid-liquid interface increased as well as the randomness at the interface. This was presumably due to the release of aqua molecules when Fe(II) or Cu(II) was adsorbed at the surface of the adsorbent.

#### 3.2.4. Effect of Fe(II) and Cu(II) Concentration

The adsorption experiments were carried out at different initial concentrations of Fe(II) and Cu(II) ranging from 10 to 400 mg·L^−1^ during 120 min and under optimal pH conditions of 5 and 6 for Fe(II) and Cu(II), respectively. The effect of the initial concentration of both tested metals on the adsorption capacity of CS-GG beads is presented in Figure 5(d). The results showed that the quantity of adsorbed metal ions increased gradually with the increase of the initial concentration of metal ions in the solution, until reaching a saturation plateau. The adsorption capacity was 126.35 mg·g^−1^ and 156.69 mg·g^−1^ for Fe(II) and Cu(II), respectively. Concerning Cu(II), the high adsorption capacity was attributed to its strong affinity for the chitosan. In fact, the increase in the initial concentration of metal ions would induce an increase in the motive force. In CS-GG beads, the sulfur, the nitrogen, and the oxygen atoms with free electrons would have a stronger interaction with metal ions.

#### 3.2.5. Adsorption Isotherm

Here, we applied adsorption isotherms to better understand the mechanism of Fe(II) and Cu(II) adsorption reactions onto CS-GG beads, such as the distribution of the adsorbent molecules between the liquid and solid phases once the equilibrium state is reached. Both models of *Langmuir* and *Freundlich* isotherms were used to determine the adsorption isotherm data from the experiments. The *Langmuir* isotherm assumed that the adsorption process occurred homogeneously on a monolayer within the adsorbent until reaching a saturation level. This model considers that all adsorption sites involved are energetically identical (homogeneous) and the intermolecular force will decrease as the distance from the adsorption surface increase [66, 67]. The isotherm of *Freundlich* was also applied, by considering that the adsorption reaction might occur on a multilayer and an energetically heterogeneous surface. However, the empirical equation of this model is suitable for high- and middle-concentration range. Therefore, this isotherm was not suitable in our study under low-concentration range, since Henry's law was not met. This isotherm permitted only to describe the nonideal and reversible adsorption.

The calculated *Langmuir* and *Freundlich* parameters were presented in Table 2. The maximum adsorption values for Fe(II) were 208.33, 212.77, and 243.90 mg·g^−1^ under 293, 3033, and 313°K, respectively. The values for Cu(II) were 217.39, 263.15, and 277.77 mg·g^−1^ under 293, 3033, and 313°K, respectively. The values of the maximum adsorption capacity (*q*_max_) obtained from *Langmuir* isotherm were much higher than the measured *q*_max_ for both Fe(II) and Cu(II). However, the adsorption capacity for Cu(II) was still higher than the one for Fe(II). In addition, *K*_*L*_ in *Langmuir* isotherm was higher for Cu(II) compared to *K*_*L*_ for Fe(II). Our results are consistent with previous studies, in which the CS showed a good affinity and selectivity for Cu(II) [68, 69]. In addition, Cu complexes with ammonia were stable, indicating that the CS was selective for this metal ion. This indicated that our beads showed better adsorption rates for Cu(II) compared to Fe(II). In addition, the higher affinity for Cu(II) can be explained by the Jahn-Teller effect, which is known to be predominant for Cu complexes [65, 70]. All of *R*_*L*_ values found were between 0 and 1 (0 < *R*_*L*_ < 1) under the studied concentration and temperature range (Figure S2 and Table 2), indicating the adsorption affinity of Fe(II) and Cu(II) onto CS-GG beads. Based on the *Freundlich* isotherm, the *K*_*F*_ value obtained for Cu(II) was higher than the value for Fe(II). A previous study showed that the tested adsorbent had a more homogeneous distribution of binding sites if the parameter *n* (or 1*/n*) value was close to 1 or even to 1 [23]. Our results showed that the values of 1*/n* obtained for both Fe(II) and Cu(II) were 0.91 and 0.70, respectively, suggesting that the binding sites were more homogeneous for the adsorption of Fe(II) and Cu(II) at the solid-liquid interface. Moreover, our data was better correlated with the *Langmuir* isotherm (*R*^2^ > 0.98) than the *Freundlich* isotherm. Therefore, our results suggested that the adsorption reactions of Fe(II) and Cu(II) did occur as a monolayer mechanism onto the CS-GG beads.

#### 3.2.6. Effect of Contact Time

Since the time to reach an adsorption equilibrium represents an important parameter, we analyzed the effect of contact time on the adsorption capacity of CS-GG beads for Fe(II) and Cu(II). The removal of Fe(II) and Cu(II) from the solution in relation to the contact time is presented in Figure 5(c). The results showed that the maximum adsorption was following the order Cu(II) > Fe(II) at all time intervals. It also showed that an increase in the agitation time improved the removal of these metal ions until reaching equilibrium after 120 min and 180 min, respectively, for Fe(II) and Cu(II). The adsorption equilibrium between the two metals was different, since Cu(II) had a greater affinity for thiol hydroxyl and amino groups [71, 72].

#### 3.2.7. Adsorption Kinetic Analysis

The kinetic rates were analyzed to provide information on the adsorption mechanism such as the rate-limiting step including the diffusion control, the chemical reaction, and the particle diffusion. To evaluate the kinetic mechanism controlling the adsorption process, the experimental data were analyzed by using the pseudo-first-order, the pseudo-second-order, and the intraparticle diffusion kinetic models. The parameters *k*_1_, *k*_2_, and *k*_*id*_ and *R*^2^ values of the kinetic models were presented in Table 3. The data plot of the pseudo-first-order model was linear with *R*^2^ < 0.99, suggesting that the adsorption of both Fe(II) and Cu(II) did not follow this model. The value of the linear regression coefficient *R*^2^ was higher than 0.99 and closer to 1 (0.99 < *R*^*2*^ < 1), which suggested that the adsorption of ions followed the pseudo-second-order model describing chemisorption. Such adsorption mechanism involved the valence forces by sharing or exchanging electrons between the metals and the adsorbent without any mass transfer in solution [23, 73].

**Figure 6:**
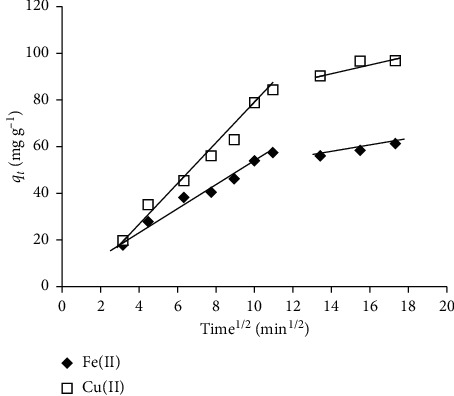
Internal diffusion indicated by the plot of q_t_ of Fe(II) and Cu(II) in relation to the time.

The results in Figure 6 presented the plots of Fe(II) and Cu(II) adsorbed per unit mass of CS-GG versus t^0.5^ (min^0.5^). The change of *q*_t_ indicated that the adsorption reaction for both metal ions had two steps. Each linear portion of the curve of *q*_t_ as a function of t^0.5^ corresponded to a step. It suggested that the first step described the adsorption process at the surface of CS-GG beads. In the second step, progressive adsorption did occur during which the intraparticle diffusion had control over the speed of the reaction [74]. However, the intraparticular diffusion would become the limiting step only if the curve did pass through the origin. In fact, the two curves (Figure 6) did not go through the origin, which might be due to the difference in the rate of mass transfer at the initial and final stages of the adsorption reaction. Moreover, it indicated that the internal diffusion was not the only step controlling the reaction speed in our study. A similar result was reported by Debnath et al. [57]. Therefore, the adsorption reaction of both Fe(II) and Cu(II) at the surface of CS-GG beads was correlated with the initial metal concentration.

#### 3.2.8. Comparative Studies

**Figure 7:**
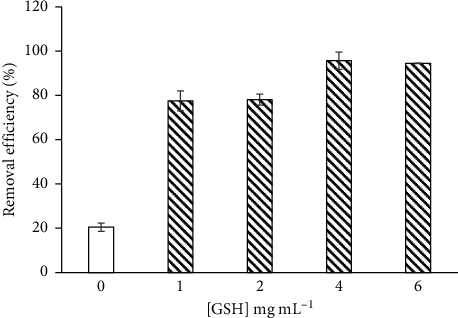
The adsorption of Fe(II) on CS-genipin (blank) and CS-GG beads (dashes) functionalized with different concentrations of GSH. The error bars represent the standard deviation of measurements for three sample runs (*n* = 3).

The adsorption capacity of CS-GG beads was dependent on its chemical structure and the number of functional groups at the surface of the polymer. Thus, we compared the maximal adsorption capacity of different CS-modified beads with our new CS-GG beads (Table 4). The maximum adsorption capacity of Fe(II) on CS-GG beads was the highest when compared to the other CS beads. In fact, this adsorption capacity was related to the concentration of GSH added to the formulation. As shown in Figure 7, a significant difference was noticed between the adsorption of metals on CS beads without GSH and CS-GG beads, which was correlated with the amount of functionalized GSH on CS-GG beads. In fact, it is well known that GSH can make complexes with metals due to the amino acid cysteine present in the *γ*-Glu-Cys-Gly structure [44, 45]. Concerning the removal of Cu(II), only the CS nanofibrils had a higher adsorption capacity than our CS-GG beads. Therefore, a comparison between our study and previous reports clearly indicated that the CS-GG beads were an effective adsorbent for the removal of Cu(II) and Fe(II) from aqueous solution.

## 4. Conclusions

In this study, we used the natural cross-linker genipin to successfully cross-link CS beads before to be functionalized with GSH. The newly formed CS-GG beads were characterized and tested as an adsorbent for the removal of Fe(II) and Cu(II) from aqueous solutions. We demonstrated that different parameters affected the adsorption reaction on the beads such as the pH, the contact time, the temperature, and the initial concentration of metals. According to the obtained results, the optimum pH for the adsorption of Fe(II) and Cu(II) was 5 and 6, respectively. In addition, the pseudo-second-order equation gave the best correlation coefficient, indicating that the chemical adsorption process was the rate-limiting step without a mass transfer in solution. The adsorption reaction of Fe(II) and Cu(II) was dependent on the temperature degree. Moreover, our results suggested that the *Langmuir* isotherm was the best fitting model with the acquired adsorption data for both tested metals than the *Freundlich* isotherm. According to the *Langmuir* isotherm, the maximum adsorption capacities of Fe(II) and Cu(II) evaluated on CS-GG beads were 208.33 and 217 mg·g^−1^, respectively. Based on the thermodynamic analysis, we showed that the adsorption process was spontaneous and endothermic. Therefore, this study demonstrated that the new synthesized CS-GG beads can be used as an efficient adsorbent for trace metals in aqueous solution. In this perspective, this new material should be further tested for the joint removal of a mixture of trace metals in solution to be applied for water decontamination.

## Figures and Tables

**Table 1:** Thermodynamic parameters for the adsorption of Fe(II) and Cu(II) ions on CS-GG beads at different temperatures.

		Fe(II)	Cu(II)
ΔH° kJ · mol^−1^		8.88	5.49
ΔG° kJ · mol^−1^	−293 K	−16.39	−17.24
	−303 K	−17.32	−18.19
	−313 K	−18.08	−18.79
ΔS° J · mol^−1^ · K^**−1**^		0.087	0.078
*R* ^2^		0.993	0.984

Experimental condition: volume of 50 mL; pH = 5 for Fe(II) and 6 for Cu(II); [Fe(II), Cu(II)] = 100 mgL^−1^; adsorbent net mass = 0.030 g; temperature = 293, 303, and 313°K; contact time = 2 h; shaking rate = 150 rpm.

**Table 2:** Parameters of *Langmuir* and *Freundlich* isotherms for the adsorption reaction of Fe(II) and Cu(II) onto CS-GG beads.

Metal ions	Langmuir					
Temp.°K	Measured *q*_max_ mg·g^−1^	*K*_*L*_ L·mg^−1^	*q*_max_ mg·g^−1^	*R_L_*	*R* ^2^
Fe(II)	293	126.4	0.0046	208.33	0.666	0.975
	303	147.5	0.0071	212.77	0.530	0.988
	313	152.3	0.0055	243.90	0.578	0.981
Cu(II)	293	156.4	0.0070	217.39	0.526	0.977
	303	189.5	0.0076	263.15	0.522	0.995
	313	199.0	0.0072	277.77	0.520	0.974

	Freundlich					
	Temp. °K	Ln K_F_	*n*		*R* ^2^	

Fe(II)	293					
	303	0.212	1.16		0.960	
	313	0.332	1.03		0.969	
Cu(II)	293	1.178	1.47		0.974	
	303	1.235	1.40		0.981	
	313	1.418	1.43		0.979	

Experimental condition: volume 50 mL; pH5 for Fe(II) and 6 for Cu(II), [Fe(II), Cu(II)] = 10–400 mgL^−1^; adsorbent net mass = 0.030 g; temperature = 293, 303, and 313°K; contact time = 2 h, shaking rate = 150 rpm.

**Table 3:** Kinetic parameters of the pseudo-first-order, the pseudo-second-order, and the internal diffusion models.

	Pseudo-first-order model				
	*k*_1_ min^−1^	*q*_e1_mg · g^−1^		*R* ^2^	
Fe(II)	0.085	60.95		0.976	
Cu(II)	0.056	82.27		0.983	

	Pseudo-second-order model				
	*k*_2_ g · mg^−1^ · min^−1^	*q*_e2_mg · g^−1^		*R* ^2^	

Fe(II)	0.6 10^−3^	66.67		0.997	
Cu(II)	0.12 10^−3^	125.00		0.994	

	Intraparticle diffusion				
	*K*_*id*_mg · g^−1^ · min^−1 0.5^	*C*mg · g^−1^		*R* ^2^	
	*K*_*id*1_	*K*_*id*2_		*R*_1_^2^	*R*_2_^2^

Fe(II)	4.906	1.344	3.596	0.990	0.991
Cu(II)	6.658	2.484	2.721	0.989	0.936

Experimental condition: volume 50 mL; pH = 5 for Fe(II) and 6 for Cu(II); [Fe(II), Cu(II)] = 100 mgL^−1^; adsorbent net mass = 0.030 g; temperature = 293°K; contact time = 10 min, during 5 h; shaking rate = 150 rpm.

**Table 4:** Maximum adsorption capacity of different modified chitosan adsorbents for Fe(II) and Cu(II) in aqueous solution.

Adsorbent	*q*_max_ (mg · g^−1^)
	Fe(II)
Chitosan cross-linked with glutaraldehyde [75]	47.25
Chitosan cross-linked with epichlorohydrin [75]	57.47
Chitosan cross-linked with ethylene glycol diglycidyl ether [75]	38.61
Mayflower seed carbon [76]	49.75
*Functionalized-glutathione on chitosan-genipin cross-linked beads* (*CS-GG*)	126.4

	Cu(II)
Chitosan nanofibrils [23]	168.7
Tannin-phenolic immobilized on cellulose [77]	55.97
Chitosan/polyvinylalcohol/polyethyleneimine membrane [78]	86.08
Magnetic-epichlorohydrin cross-linked chitosan [14]	123.10
Chitosan cross-linked with epichlorohydrin-triphosphate [79]	130.72
*Functionalized-glutathione on chitosan-genipin cross-linked beads* (*CS-GG*)	156.4

## Data Availability

The datasets analyzed in this manuscript are available on request to the corresponding author.

## Supplementary Materials

Supplementary Materials Figure S1: thermodynamic plots of −ΔG° in relation to temperature (°K). Figure S2: *Langmuir* plots of experimental data of *C*_e_/*q*_e_ versus *C*_e_ for Fe(II) and Cu(II) adsorption on CS-GG beads. Figure S3: *Freundlich* plots of experimental data showing Ln *q*_e_ versus Ln *C*_e_ for the adsorption of Fe(II) and Cu(II) on CS-GG beads. Figure S4: pseudo-first-order of the adsorption of Fe(II) (a) and Cu(II) (b) on CS-GG beads. Figure S5: pseudo-second-order of the adsorption of Fe(II) (a) and Cu(II) (b) on CS-GG beads.
