# Supplementary material for: Functionalized Glutathione on Chitosan-Genipin Cross-Linked Beads Used for the Removal of Trace Metals from Water
Source: Int J Biomater. 2020 Sep 14;2020:4158086. doi: 10.1155/2020/4158086 (PMC7509577; doi:10.1155/2020/4158086)
Supplement: Supplementary Materials — Figure S1: thermodynamic plots of −ΔG° in relation to temperature (°K). Figure S2: Langmuir plots of experimental data of Ce/qe versus Ce for Fe(II) and Cu(II) adsorption on CS-GG beads. Figure S3: Freundlich plots of experimental data showing Ln qe versus Ln Ce for the adsorption of Fe(II) and Cu(II) on CS-GG beads. Figure S4: pseudo-first-order of the adsorption of Fe(II) (a) and Cu(II) (b) on CS-GG beads. Figure S5: pseudo-second-order of the adsorption of Fe(II) (a) and Cu(II) (b) on CS-GG beads. [file 4158086.f1.zip › 4158086.f1/Fig_S2.docx]

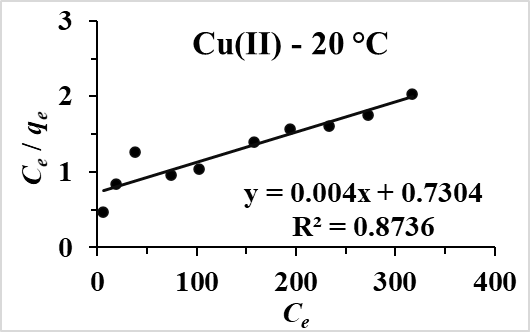

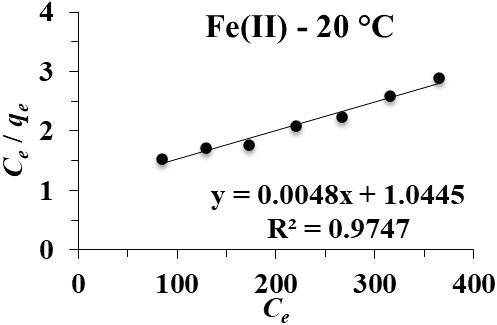

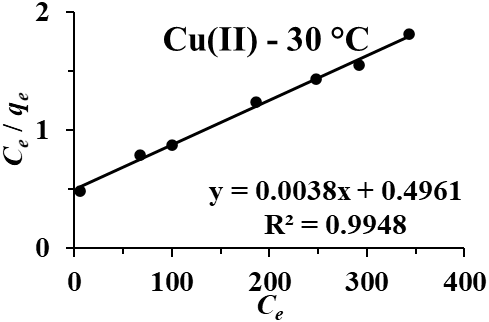

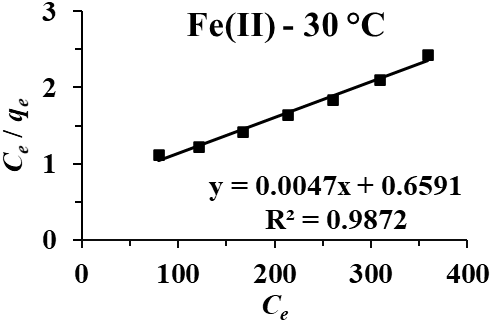

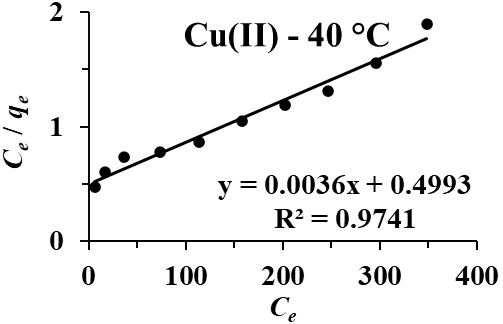

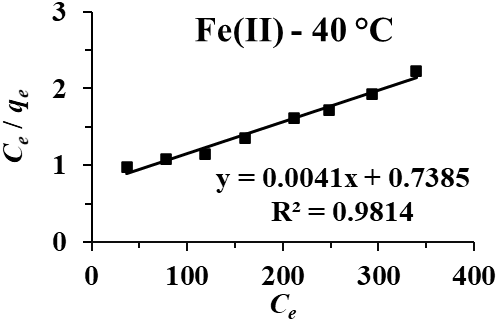


Figure S2: *Langmuir* plots of experimental data of C_e_/q_e_ versus C_e_ for Fe(II) and Cu(II) adsorption on CS-GG beads.
